# Visits to Private Asylums

**Published:** 1904-05-28

**Authors:** 


### VISITS TO PRIVATE ASYLUMS.

By Our Rambling Reporter.

### PERITEAU, WINCHELSEA, SUSSEX.

This is a delightful old-fashioned residence in the pretty little town of Winchelsea, one of the Cinque ports, and now looking at her sister Rye over meadows and marshes where once rolled the sea. The little town, or rather village, is most peaceful and even the railway station is three-quarters of a mile away, as if the scream of the locomotive should not be heard in it, or if heard at all it must be far enough off not to seriously break the silence.

Periteau is actually in the town and placed at the corner of one of the pretty streets, but nevertheless it is quite private, and its acre of garden is so secluded that one might fancy there was no other habitation within speaking distance. After being inspected in the year 1882 by the Commissioners in Lunacy, the house was licensed for the reception of five insane lady patients, and ever since then it has been owned and managed by the present proprietress.

We saw no locked doors, and all the rooms are exactly what would be found in a well-furnished and carefully kept house of similar size not intended for patients at all. It is the type of private asylum which we should like to see more numerously represented than it is at present. The dining-room and drawing-room are of fair size, and the latter opens by French windows on to the lawn where are shady walks and a large summer-house. The bedrooms are equally nice, and each lady has a room to herself, or rather she shares the room with her own lady-companion. There are as many lady-companions as there are patients, hence the latter are well looked after and have ever at hand one of their own sex and position with whom they can associate and in whom they can confide. The patients have all their meals, even their late dinner, with the proprietress, and everything is conducted on the most liberal scale.

The patients are taken away every year for five or six weeks; and however nice Periteau may be such annual change is a desirable thing in the interests of the curable and incurable alike. The establishment is in every way admirably suited for the treatment of quiet cases. Vacancies do not occur very often, and as might be expected the fees are rather high.

